# The clouded debate: A systematic review of comparative longitudinal studies examining the impact of recreational cannabis legalization on key public health outcomes

**DOI:** 10.3389/fpsyt.2022.1060656

**Published:** 2023-01-11

**Authors:** Maria Athanassiou, Alexandre Dumais, Inès Zouaoui, Stéphane Potvin

**Affiliations:** ^1^Centre de recherche de l'Institut Universitaire en Santé Mentale de Montréal, Montreal, QC, Canada; ^2^Department of Psychiatry and Addiction, Faculty of Medicine, University of Montreal, Montreal, QC, Canada; ^3^Philippe-Pinel National Institute of Legal Psychiatry, Montreal, QC, Canada

**Keywords:** cannabis, legalization, recreational, review, longitudinal

## Abstract

**Background:**

Ineffective cannabis regulatory frameworks such as prohibition have sparked interest in alternative solutions to reduce individual and societal harms. While it has been suggested that the recreational legalization process has yielded early successes, the relatively recent implementation of the novel policies has provided a modest time frame for a truly thorough establishment and assessment of key population-level indicators. The following systematic review focuses on identifying the downstream public health sequelae of cannabis legalization policies, including parameters such as cannabis consumption rates, hospitalization rates, vehicular accidents and fatalities, criminal activity, and suicidal behaviors, as well as other substance use trends.

**Methods:**

An exhaustive search of the MEDLINE and Google Scholar databases were performed to identify high-quality (1) longitudinal studies, which (2) compared key public health outcomes between regions which had and had not implemented recreational cannabis legalization (RML) policies, (3) using distinct databases and/or time frames. Thirty-two original research articles were retained for review.

**Results:**

Adult past-month cannabis consumption (26+ years) seems to have significantly increased following RML, whereas young adult (18–26 years) and adolescent (12–17 years) populations do not show a significant rise in past-month cannabis use. RML shows preliminary trends in increasing service use (such as hospitalizations, emergency department visits, or poisonings) or vehicular traffic fatalities. Preliminary evidence suggests that RML is related to potential increases in serious/violent crimes, and heterogeneous effects on suicidal behaviors. While the research does not illustrate that RML is linked to changing consumptions patterns of cigarette, stimulant, or opioid use, alcohol use may be on the rise, and opioid prescribing patterns are shown to be significantly correlated with RML.

**Conclusion:**

The current data supports the notion that RML is correlated with altered cannabis consumption in adults, potentially increased criminal activity, and a decline in opioid quantities and prescriptions provided to patients. Future work should address additional knowledge gaps for vulnerable populations, such as individuals with mental health problems or persons consuming cannabis frequently/at higher THC doses. The effects of varying legalization models should also be evaluated for their potentially differing impacts on population-level outcomes.

## 1. Introduction

It has been suggested that international cannabis prohibition mandates have failed to achieve key goals such as harm reduction, increased prevention and treatment, and have instead generated negative consequences, including increased contributions toward global disease burden over time (1, 2) and exacerbating social inequalities through disproportionate impacts on people of color (3, 4). As such, ineffective regulatory frameworks such as cannabis prohibition have sparked interest in alternative solutions to reduce individual and societal harms (5). Recently, countries such as Uruguay, Canada, and certain states of the United States have enacted recreational marijuana laws (RML). While the overarching frameworks vary between locations, certain RML laws propose to enhance the protection of vulnerable populations; strengthen health education programs; provide access to quality-controlled cannabis; and enable the close monitoring of public health outcomes through these new regulatory frameworks (6). Despite these beneficial aspirations, the enactment of cannabis legalization policies remains hotly questioned. Several thought leaders have denoted an opposition against the hasty implementation of legalization policies, warning against the escalation of use and related harms among the most vulnerable populations, such as youth (7), an increase in driving under the influence, or increased risk of using other drugs, including harder drugs (8). Despite having collected close to a decade of research evidence, we have yet to determine unequivocal findings to support either side of the discussion relating to recreational cannabis legalization laws.

Regarding the impacts of RML on general cannabis consumption, studies conducted in several states across the US have found discrepant results. Initial evidence in adult populations have found increases in cannabis use over time (9–11), decreases of use (12), or even a lack of change altogether (13). Youth populations also demonstrate varying effects, with evidence for overall exacerbated use (14), diminished use (15), or show no impact (16). Of importance, the largest source of data collected on consumption metrics relate to past month cannabis use, few have investigated frequent use, and sparse have examined trends in cannabis use disorder. As marijuana consumption trends may vary over time, using outcome metrics such as past month marijuana use may not provide an accurate reflection of true individual consumption trends over time. This may entail an over or under-estimation in the number of individuals at highest risk of adverse health consequences associated with cannabis use.

Beyond simple consumption patterns, several other population parameters have been monitored over time to determine the impacts of RML. Seminal work developed by Lake and colleagues highlighted the use of 28 indicators to monitor RML effects, including public safety measures such as vehicle injuries/fatalities and crimes; other substance use and overdose trends; and hospitalizations related to cannabis use (17). Research has suggested potential surges in vehicular fatalities and crimes–specifically, increases in crimes such as burglary, larceny, violent assaults, and so forth (18, 19). Preliminary evidence points to potential increases in healthcare service use related to cannabis (20–22). However, these initial assumptions seem to be skewed by an overrepresentation of increases in specific states, such as Colorado. Other substance use, such as alcohol, tobacco, or illicit drugs use, has seen trends of increases (23), decreases (11), and no changes (24).

The discrepant results in the current literature can be partially attributed to the methodology and sampling used in the research studies. Most are performed in a single location, thus omitting trends over the same time course in a comparator location. Thus, such studies may highlight changes that are not necessarily related to legalization *per se*, but may actually reflect other unspecific factors, such as the perception of harms, for instance. Other studies have used a comparator location but have collected data only post-legalization. These are both critical methodological aspects to consider, as certain locations may be already experiencing upticks in cannabis consumption prior to legalization, thus post-legalization patterns across regions should be interpreted with caution. Certain studies collect only a single datapoint prior to RML implementation, or only a single datapoint post RML implementation, providing little information on the trends already occurring prior to RML implementation, as well as little information into long-term effects if studying a short post-RML period. Considering the limitations of studies using these methodological strategies, it would be beneficial to update the current state of the knowledge of RML impacts on population health metrics using longitudinal comparative studies.

As such, the following systematic review seeks to shed light on the clouded debate of the impacts of RML on key public health metrics. Importantly, we aim to perform a systematic review of studies which will provide a high level of insight: research articles which follow RML and non-RML states, with a baseline assessment of public health trends prior to RML implementation. This systematic review will focus on key metrics outlined by Lake et al. (17) to examine if RML implementation affects youth/young adult/adult cannabis consumption, service use, vehicular crashes/fatalities, crimes (unrelated to cannabis possession), and other drug use. The evidence provided in this review will help provide recommendations for future cannabis legalization policy research.

## 2. Methods

### 2.1. Search strategy

The search strategy was completed in accordance with the Preferred Reporting Items for Systematic Reviews and Meta-Analyses (PRISMA) standards (25). Potential articles were discovered through an exhaustive search of the MEDLINE database and Google Scholar for studies expanding from January 1, 2012–which corresponds to the year where recreational cannabis was legalized for the first time in Colorado and Washington–until February 1, 2022. The following terms were employed to direct our search for research articles: (“marijuana/marihuana,” “cannabis,” “illicit), the independent factor (“legalization,” “recreational”) and the outcomes of interest (“use,” “consumption,” “hospital^*^”, “traffic,” “crime,” “alcohol,” “stimulant,” “opioid,” “nicotine”). Cross-referencing of previous systematic reviews on the topic was also performed.

### 2.2. Eligibility criteria

Longitudinal observational studies were retained for the purposes of this systematic review. Specifically, we retained studies that: (i) had a baseline assessment (pre) prior to the implementation of recreational cannabis legalization, and a subsequent assessment (post) at least 6 months after the implementation of RML.; and (ii) which also longitudinally assessed at least one comparator location (control) which did not undergo RML. Of note, in some article, the same subjects were investigated over time, while in others, multiple measures were acquired over time in different samples of persons living within a state.

#### 2.2.1. Exclusion criteria

In addition to the above-referenced criteria, studies were excluded if they evaluated medical cannabis legalization. We omitted studies which focused on solely on the impact of recreational cannabis legalization on arrests for possession of cannabis. Studies in languages other than English were also excluded. We did not retain studies that lacked a comparison group, or studies that did not have at least one pre-legalization evaluation and one post-legalization evaluation. There was an important number of publications which utilized overlapping databases and/or time points to study the effects of RML. As such, for all overlapping research initiatives, M.A. and S.P. identified the studies used for primary analyses purposes, which provided the latest data, included the highest number of participants and/or the highest number of states, and provided the longest follow-up period. Any overlapping studies which investigated single locations were retained for secondary analysis purposes when these studies reported data on specific outcomes that had not been reported in the primary analyses (example: specific effects in particular locations). The final decision on the inclusion and exclusion of studies was determined by consensus between M.A. and S.P.

### 2.3. Data extraction and quality assessment

The following information was extracted by two independent authors (M.A. and I.Z.): (1) type of population studied (including sample size (if available), average age, sex ratio); (2) RML locations studied; (3) non-RML comparator locations; (4) years assessed; (5) data source; (6) outcome measures analyzed; (7) confounding factors controlled for/considered in the analyses; (8) statistical models used in the analyses; (9) overarching results.

A quality assessment was then performed on the retained articles using an adapted version of the Newcastle - Ottawa Quality Assessment Scale for cohort studies (26). Briefly, studies were rated on strength of sampling selection, comparability, outcome, and follow-up time. As per the tool, studies were rated using a 3-point system (0–2 points), and accumulated scores on the 7 rated items qualified them as having either weak (0–4), moderate (5–9) or strong (10–14) reporting strength.

## 3. Results

### 3.1. Study selection

**Figure 1:**
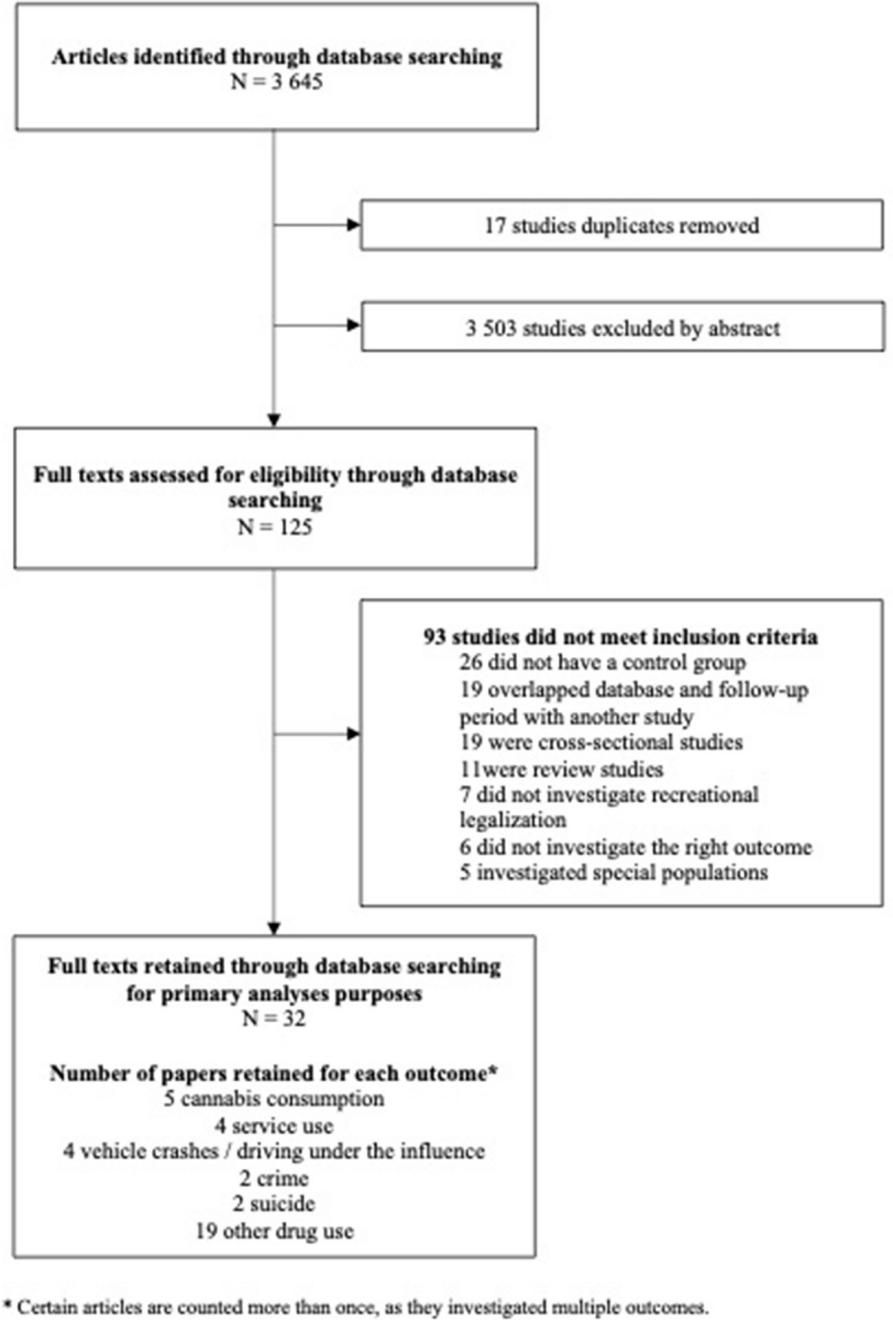
PRISMA flow Chart for the impact of the legalization of recreational cannabis on key public health outcomes.

Out of 3645 studies identified in the database search, 125 articles underwent full-text screening, whereby 93 articles were excluded, predominantly because they were not longitudinal in nature (26), did not include a comparator group (19), or the database and time frame used in the analysis overlapped with another study retained for review (20). Thirty-two unique articles stemming from this database search were included in the primary analyses (Figure 1 PRISMA flow chart).

### 3.2. Study characteristics

Studies investigating the effects of cannabis legalization on health outcomes are summarized in Table 1. Most studies included in the review investigated multiple locations which had or had not implemented RML. While most studies accounted for a large number of covariates, including age, sex, socioeconomic status, prior education, and prescription drug monitoring programs, it should be noted that a few publications failed to account for more than a few basic confounders.

**Table 1:** Overview of longitudinal studies investigating the impact of cannabis legalization public health outcomes used for primary analyses.

**Study**	**Year**	**Population**	**Location (s) studied**	**Comparator location (s)**	**Years assessed**	**Data source**	**Outcome (s) of interest**	**Ascertainment of outcome (s) of interest**	**Brief main findings of the impacts of RML**
Alcocer	2020	All persons in participating states	CO	32 non-RML states	1999–2017	WONDER	Opioid mortality	Opioid overdose mortality rate per 100,000 in population	No significant difference
Alley	2020	18–26-year-old college students	All RML states	All non-RML states	2008–2018	NCHA-II	Other drug use	Self-reported use	Decreased odds of binge drinking, no significant difference for other drug use
Bae	2019	18–26-year-old college students	7 RML states	41 non-RML states	2008–2018	NCHA-II	Cannabis use	Self-reported past-month use (any), frequent use (>20 uses in last month)	Increased odds of cannabis use
Benedetti	2021	Adult drivers	All RML states	All non-RML states	2013-2017	TSCI	Driving under the influence of cannabis	Self-reported past-year driving within 1 h of marijuana use	No significant difference
Bhave	2020	All persons in participating states	CO, OR, NV, WA	Synthetic control	2012–2017	Retail scanner data from A.C. Nielsen	Nicotine use	Weekly cigarette sales in packs	Increased odds of nicotine use
Cerdà	2020	Persons 12+ in participating states	CO, WA, AK, OR	All non-RML states	2008–2016	NSDUH	Frequent use in the past month, past-year CUD overall	Self-reported past-month use, frequent use (>20 days or more of use in the past month), past-year prevalence of CUD (instrument that assessed symptoms corresponding to DSM-IV criteria)	Group 12–17 years: No increase in frequent use; increased past-year CUD prevalence Group 18–26 years: No difference for any outcome Group 26+ years: Increase in both outcomes
Cerdà	2017	High school students	CO, WA	45 non-RML states	2010–2015	MTF	Cannabis use	Self-reported past-month use (any)	Increased odds of cannabis use in youth in Washington (grades 8–10)
Chan	2020	All persons in participating states	All RML states	Non-RML states	1999–2017	NVSS	Opioid mortality	Means of opioid mortality rates (per 100,000 population)	Decreased odds of opioid mortality
Coley	2021	Youth	6 RML states	Non-RML states	2015 and 2017	YRBS	Cannabis use, other drug use	Past-month marijuana, alcohol, cigarette, e-cigarette use (number of times)	No significant difference for cannabis use, small increased odds of cigarette use
Delling	2019	Inpatients in participating states	CO	NY, OK	2010–2014	HCUP	Service use	Total number of hospitalizations, length of inpatient stay, healthcare costs, hospitalization related to multi vehicle collisions	Increased odds of cannabis-related service use
Doucette	2021	All persons in participating states	CO, WA	Synthetic control	2000–2018	NCHS	Suicide rate	Annual, state-level deaths by suicide	Increased odds of death by suicide in Washington, no significant difference in Colorado
Drake	2021	All persons in participating states	CA, ME, MA, NV	Non-RML states	2011–2017	HCUP	Service use	Log opioid-related ED visit rates per 100,000 population in states	No significant difference in service use
Kamer	2020	All persons in participating states	CO, WA, OR, and AK	20 non-RML states	2008–2018	FARS	Traffic fatality rates	Traffic fatality rates	Increased odds of traffic fatalities
Kerr	2017	18–26-year-old college students	OR	6 non-RML states	2014 and 2016	HMS	Cannabis use, other drug use	Self-reported past-month use (any) of marijuana, cigarette and frequency of heavy alcohol	Increased odds of cannabis use only in recent heavy alcohol users
Kim	2021	All persons in participating states	AK, CA, CO, DC, MA, ME, NV, OR, WA	Non-RML states	2004–2017	NSDUH	Cannabis use, alcohol use	Self-reported past month marijuana use (any), past month alcohol use (any)	Increased odds of cannabis use only in adults
Kropp Lopez	2020	All persons in participating states	CO	UT, MD	2007–2017	DEA ARCOS	Opioid prescriptions	Prescription opioid distribution for OUD treatment (oral morphine milligram equivalents)	Significantly increased oral MME
Lane	2019	All persons in participating states	CO, WA, OR	AL, AR, FL, GA, IN, IA, KY, MI, MS, MO, ND, NC, SC, SD, TN, TX, VA, WV, WI	2009–2016	WONDER	Traffic fatalities	Monthly traffic fatalities rates per million residents	Decreased odds of traffic fatalities only in Washington
Lopez	2021	Medicaid enrollees in participating states	AK, CA, CO, DC, MA, ME, NV, OR, WA	Non-RML states	2013–2017	Medicare Part D Prescription Drug Event database	Opioid prescriptions by orthopedic surgeons	Annual aggregate daily doses of all opioid medications (excluding buprenorphine) prescribed by orthopedic surgeons in each US state (and DC)	No association between RML and opioid prescriptions
Lu	2018	All persons in participating states	CO, WA	Non-RML states	1999–2016	FBI's UCR	Crimes	Monthly crime rates: violent, property, aggravated assault, auto theft, burglary, larceny, and robbery rates	No significant difference for violent crimes, only short-term increase in property crimes in Colorado
Lu	2020	All Medicaid enrollees in participating states	All RML states	Non-RML states	2005–2019	Consumer Expenditure Interview Survey	Alcohol use	Alcohol expenditures	Increase in alcohol use
Masonbrink	2021	Youth	CA, CO, DC, MA, WA	AZ, CT, DE, FL, IL, IN, MD, MN, MO, NJ, NY, OH, PA, UT	2008–2019	Inpatient Essentials database	Service use	Annual incidence of a hospitalization with a cannabis-related diagnosis (i.e., cannabis-related hospitalization)	Increased odds of service use
Matthay	2021	All Medicare enrollees in participating states	All RML states	All non-RML states	2003–2017	Clinformatics Data Mart; Optum Inc	Self-harm, Crimes	Claims for self-harm and assault injuries based on International Classification of Diseases codes	Increased odds of self-harm injury in males <21 years old, increased odds of physical assault for males and females <21 years old
McMichael	2020	Patients at outpatient pharmacies in participating states	All RML states	MCL and no marijuana law states	2011–2018	Symphony Health's IDV^®^ dataset	Opioid prescriptions	(1) the total number of MMEs prescribed by each provider, (2) the total days' supply prescribed by each provider, (3) the number of unique patients to whom each provider prescribed opioids, (4) the percentage of a provider's patients receiving any opioids, and (5) whether a provider prescribed any opioids.	Significantly decreased oral MME
Mennis	2021a	All young adults in participating states	All RML states	All non-RML states	2008–2017	SAMHSA (TEDS-A), NSDUH	Service use	Treatment admissions for cannabis (number of young adult's treatment admissions/young adult population),	Decreased odds of service use
Mennis	2021b	Youth	CO, WA	Non-RML states	2008–2017	SAMHSA TEDS-A	Service use	Mean observed treatment admissions rates (per 10,000 population)	Increased odds of service use in Colorado
Miller	2017	18–26-year-old college students	WA	National average	2005–2015	NCHA, NSDUH	Cannabis use, other drug use	Self-reported past-month use	Increased odds of cannabis use, no significant difference for other drugs
Shi	2020	All persons in participating states	All RML states	Non-RML states	2010–2017	USNPDS	Service use	Cannabis exposures reported to the US National Poison Data System	Increased odds of service use (unintentional exposures and exposures without medical consequences)
Shi	2019	All Medicaid enrollees in participating states	CO, WA, AK, DC, OR, CA, MA, ME, NV	HI, MI, MT, NM, RI, VT	2010–2017	Medicaid State Drug Utilization Data	Opioid prescriptions	(1) Number of opioid prescriptions, (2) Total doses of opioid prescriptions (in quantity of MME)	Significantly decreased oral MME
Veligati	2020	All persons in participating states	All RML states	All non-RML states	1990–2016	NIAAA, AEDS, Tax Burden on Tobacco	Other drug use	Per capita consumption of alcohol and cigarettes as measured by state tax receipts	No significant difference
Wallace	2020	18–26-year-old college students	CO	National Average	2011–2015	NCHA	Cannabis use	Self-reported 30-day use of cannabis	Increased odds of cannabis use
Weinberger	2022	All persons in participating states	All RML states	Non-RML states	2004–2017	NSDUH	Cannabis use, nicotine use	Self-reported past-month cannabis use (any)	Decreased odds of cannabis use in youth, increased odds of cannabis use in adults, decreased odds of nicotine use in youth
Wen	2021	Patients with employer- sponsored health insurance	All RML states	Non-RML states	2009–2015	Truven Health MarketScan Commercial Claims and Encounters Database	Opioid prescriptions	Monthly MME per enrollee	Significant decreased of oral MME

RML, Recreational Marijuana Legalization; WONDER, Wide-ranging ONline Data for Epidemiologic Research; NCHA, National College Health Assessment; TSCI, Traffic Safety Culture Index; MTF, Monitoring the Future; NVSS, National Vital Statistics System; YRBS, Youth Risk Behavior Surveillance; HCUP, Healthcare Utilization Project; NCHS, National Center for Health Statistics; FARS, Fatality Analysis Reporting System; HMS, Healthy Minds Study; NSDUH, National Survey on Drug Use and Health; DEA ARCOS, Drug Enforcement Administration Automation of Reports and Consolidated Orders System; FBI's UCR, Federal Bureau of Investigation Uniform Crime Reporting; SAMHSA TEDS-A, Substance Abuse and Mental Health Services Administration Treatment Episode Data Set; USNPDS, US National Poison Data System; NIAAA, National Institute on Alcohol Abuse and Alcoholism; AEDS, Alcohol Epidemiologic Data System; MME, milligram morphine equivalent.

### 3.3. Study quality and reporting strength

As described in Table 2, the range of scores from the extracted articles varied greatly (between 5 and 12), with an average overall strong quality score of 10.25. As a quality score of 10 and above is considered as methodologically robust, 25 studies of the total 32 were deemed as good-quality evidence to accurately depict the relationships between RML and the selected population-level outcomes. Overall, the selected samples were representative of the targeted population; the nature of the studies yielded large sample sizes including thousands of persons. The intervention and comparator locations were clearly defined and represented a large number of states. The outcomes included relatively objective observable outcomes, mostly from government-mandated databases. A wide range in the years assessed was noted, whereby studies had a follow-up period of 4–18 years post-baseline assessment.

**Table 2:** Bias assessment of longitudinal studies investigating the impact of cannabis legalization public health outcomes used for primary analyses.

**First Author**	**Year**	**Representativeness**	**Sample Size**	**Non-respondents**	**Ascertainment**	**Comparability**	**Assess outcome**	**Statistical test**	**Follow-up time frame**	**Score**
Alcocer	2020	1	1	1	2	2	2	1	2	12
Alley	2020	1	1	0	2	2	2	1	2	11
Bae	2019	1	1	1	2	2	2	1	2	12
Benedetti	2021	1	1	1	2	2	2	1	1	11
Bhave	2020	1	1	0	2	2	2	1	1	10
Cerdà	2020	1	1	1	2	2	2	1	2	12
Cerdà	2017	1	1	1	2	2	2	1	1	11
Chan	2020	1	1	1	2	2	1	1	2	10
Coley	2021	1	1	1	2	2	2	1	0	10
Delling	2019	1	1	1	2	2	2	1	1	11
Doucette	2021	1	1	1	2	2	2	1	2	12
Drake	2021	1	1	1	2	1	0	1	1	8
Kamer	2020	1	1	0	2	1	2	1	2	10
Kerr	2017	1	1	1	2	2	2	1	0	10
Kim	2021	1	1	1	2	2	2	1	2	12
Kropp Lopez	2020	1	1	1	1	0	2	0	2	8
Lane	2019	1	1	0	2	0	2	1	1	8
Lopez	2021	1	1	0	1	2	2	1	1	9
Lu	2018	1	1	1	2	0	2	1	2	10
Lu	2020	1	1	1	2	1	2	1	2	11
Masonbrink	2021	1	1	1	2	2	2	1	2	12
Matthay	2021	1	1	1	2	2	2	1	2	12
McMichael	2020	1	1	1	2	1	2	1	1	10
Mennis	2021b	1	1	1	1	1	2	1	1	9
Mennis	2021a	1	1	1	2	1	2	1	1	10
Miller	2017	1	1	0	2	2	2	1	2	11
Shi	2020	1	1	1	2	2	2	1	1	11
Shi	2019	1	1	1	2	2	2	1	1	11
Veligati	2020	1	1	1	2	2	2	1	2	12
Wallace	2020	1	1	0	1	0	1	1	0	5
Weinberger	2022	1	1	1	2	1	2	1	2	11
Wen	2021	1	1	1	1	1	1	1	1	8

### 3.4. Results of study outcomes

#### 3.4.1. Adult consumption

One research article examined the impact of RML on adult (26 years+) past-month cannabis use (11). Using the National Survey on Drug Use and Health (NSDUH) across 11 states. The authors determined that RML was associated with an increase in past-month adult consumption. One study evaluated past-month frequent use, as well as past-year CUD prevalence in adults using the NSDUH across 4 RML locations: Colorado, Washington, Alaska, and Oregon (10). The data yielded a significant increase in past-month frequent use (from 2.13 to 2.62%), as well as an increase in past-year CUD (from 0.90 to 1.23%).

#### 3.4.2. Young adult (18–26) consumption

Three publications assessed the impact of recreational marijuana legalization on young adult past-month cannabis use in multiple RML states (9, 11, 27). While two studies demonstrate a lack of effect of RML, Bae and Kerr (9) found that college students in states with legalized recreational cannabis use had an increased prevalence of past-month use [adjusted Odds Rations (OR) of 1.23]. Three additional studies were retained for secondary analysis purposes and investigated the effects of RML specifically in in Colorado, Oregon, and Washington (28–30). In all three cases, RML was linked to increased past-month cannabis use in young adults. Regarding frequent use, Bae and Kerr (9) has found an increased adjusted Odds Rations (OR) of 1.18, whereas Cerdà et al. (10) failed to find evidence of increased past-month frequent use, or past-year prevalence of CUD, among young adults.

#### 3.4.3. Youth (12–17)

Three primary articles investigated past-month cannabis use in adolescents (1, 31, 32). While Kim et al. (11) found a decrease in past month use, Coley et al. (32) did not find evidence for an increase or decrease in use, and Cerdà et al. (31) only found increases in past-month use of eighth and tenth graders. One additional study was retained for secondary analysis purposes and demonstrated that RML was associated with heightened past-month use in Alaskan youth (17). Cerdà et al. (10) did not report increases in past-month frequent use, however, did denote an increase in past-year CUD prevalence in youth (OR 1.25; 95%, confidence interval (CI) 1.01–1.55).

#### 3.4.4. Healthcare-related service use

Four articles studied RML effects and service use, including cannabis-related hospitalizations, emergency department visits and reported cannabis exposures (33–36). Three studies denoted heightened service use in association with RML status, whereas Mennis et al. (37) found a decrease in cannabis-related treatments admissions in young adults in seven legalized states.

#### 3.4.5. Multi-vehicle collisions, traffic fatalities or driving under the influence

Four studies assessed traffic-related accidents, injuries, and driving while intoxicated according to recreational legalization status. Delling et al. (33) extracted multi-vehicle collision data from the Healthcare Cost and Utilization Project database for the state of Colorado and found a significant impact of RML. These findings were echoed by Kamer et al. (38), who used Fatality Analysis Reporting System (FARS) data to demonstrate a link between a doubling in traffic fatality rates and RML in Colorado, Washington, Oregon, and Alaska. Lane et al. (39) utilized the Centers for Disease Control and Prevention Wide-ranging ONline Data for Epidemiologic Research (CDC WONDER) database to show that Washington state experienced an increase in traffic fatalities, whereas Colorado and Oregon did not. While no information on location is provided, Benedetti et al. (40) extracted data from the Traffic Safety Culture Index (TSCI) and found no effect of RML status on driving while under the influence.

#### 3.4.6. Crime

Two studies evaluated the effects of RML on crimes excluding arrests for marijuana possession. Lu et al. (18) reviewed data extracted from the Federal Bureau of Investigation's Uniform Crime Reporting Program for the states of Colorado and Washington. Between 1999 and 2016, the authors concluded that violent crimes did not significantly increase in either state due to RML, however certain property crimes rates were significantly heightened post-legalization. In Colorado, larceny seemed to drive property crime rate increases, whereas in Washington, rates of burglaries and aggravated assaults were predominantly affected. As well, Matthay et al. (41) used Clinformatics data to determine that RML status was associated was linked significant increases in assaults of persons younger than 21 years of age.

#### 3.4.7. Alcohol use

A series of seven articles investigated the association between recreational cannabis legalization and alcohol use. Three studies provide evidence for an increase in alcohol consumption in RML states, as reported by the Consumer Expenditure Interview Survey (42), HCUP data (33), and the ACHA-National College Health Assessment II (NCHA-II) (23), across RML states. Curiously, 3 studies failed to show an association between legalization and alcohol use (24, 27, 32). One research article demonstrated a decrease in alcohol use following legalization in Colorado across 11 US RML states (11).

#### 3.4.8. Cigarette use

Six studies were retained to evaluate cigarette consumption in response to RML implementation in the US. Most studies failed to find an effect of recreational legalization on tobacco use, as per data derived from the Healthy Minds Study database in Oregon youth/young adults (27), the NCHA-II in several RML states (23), the Tax Burden on Tobacco data (24), and the Youth Risk Behavior Surveys (YRBS) in six RML locations (32). Nonetheless, longitudinal data has also found evidence of potential increases in cigarette use in Colorado, Oregon, Nevada, and Washington (43), as well as decreases in use in almost 10 states (15).

#### 3.4.9. Opioid metrics

##### 3.4.9.1. Opioid use

One original research article evaluated the impact of RML on self-reported opioid use. Alley et al. (23) collected responses to self-reported past-month opioid use from the NCHA-II from over 800 000 college students and determined that legalization status was not associated with opioid consumption in young adults (23).

##### 3.4.9.2. Opioid-related service use

Two research studies assessed the effects of RML on opioid-related service use. Drake et al. (44) examined the opioid-related emergency department visit rates per 100,000 population in California, Maine, Massachusetts, and Nevada using the Healthcare Cost and Utilization Project (HCUP) database. While they found initial decreases in opioid-related ED visits in RML states, the effects were abolished by the end of the study period (44). Mennis et al. (37) explored the impact of RML in adolescents and young adults (12–24 years of age) in Washington and Colorado compared to non-RML states regarding opioid-related treatment admission from the Substance Abuse and Mental Health Services Administration (SAMHSA) Treatment Episode Dataset–Admissions database. The first difference-in-difference analyses determined that RML was not linked to treatment admissions. However, when analyzed separately, Colorado yielded a significant increase in opioid-related treatment admissions, while Washington demonstrated a significant decrease in opioid-related treatment admissions (37). In sum, the current data do not provide sufficient evidence to support the notion that RML is correlated with alterations in opioid-related service use.

##### 3.4.9.3. Opioid prescriptions

Five studies assessed the impact of RML on opioid prescriptions. McMichael et al. (45) collected information from over 1 billion individual prescriptions derived from the Symphony Health's IDV^®^ (Integrated Dataverse) dataset of patients of outpatient pharmacies in all RML and non-RML states. Using difference-in-difference analyses, the authors determined that recreational cannabis legalization corresponded with a significant decrease in the quantity of opioids (in morphine milligram equivalents or MMEs) prescribed to patients (45). This significant finding was echoed throughout three of the other four studies which investigated MMEs as their main outcome of interest. Wen et al. (46) retrieved MME data from patients with employer-sponsored health insurance between RML and non-RML states and found a significant 13% reduction in monthly MMEs in RML state patients (46). Shi et al. (47) extracted MME doses for Medicaid patients of RML vs. non RML states using the Medicaid State Drug Utilization Data and yielded total MME dose reductions for Schedule 3 opioids by 30% in RML states (47). Lopez et al. (48) used an indirect measure to investigate opioid use, through prescription opioid distribution numbers for opioid use disorder treatment (in MME equivalents) and found a reduction in this outcome in Colorado and Maryland–but not for the state of Utah. Only one study failed to establish a significant association between recreational cannabis legislation and MMEs (48). Shi et al. (47) also determined that the average number of opioid prescriptions written by physicians declined by 32% with the implementation of recreational cannabis legalization policies. In sum, the current data supports the notion that RML is correlated with a change in opioid prescription practices, including a reduction of average MMEs prescribed to patients, as well as the number of prescriptions provided to patients.

##### 3.4.9.4. Opioid-related deaths

One study observed trends in opioid-related deaths pre and post recreational cannabis legalization. Alcocer et al. (49) investigated a wide temporal range (1999–2017) in Colorado and extracted data from the CDC's WONDER database and found no evidence of RML effects on opioid overdose mortality rates per 100,000 population when compared to a synthetic control model (i.e., pooled data from multiple donor states to provide an accurate comparator) (49).

#### 3.4.10. Suicide

Two studies evaluated the effects of RML on deaths by suicide. Matthay et al. (41) examined claims for self-harm injuries based on International Classification of Diseases codes from all RML states using the Clinformatics Data Mart. The analyses yielded a significant association between heightened rates of self-injury for your males in states that had legalized recreational cannabis (41). Doucette et al. (50) performed a more restricted analysis on data derived from Washington State and Colorado and found heterogeneous effects of RML. Specifically, Washington state youth and young adults demonstrated a link between deaths by suicide and RML status, whereas Colorado residents did not (50).

## 4. Discussion

The following review aimed to evaluate the evidence linking population-level health metrics with the implementation of recreational legalization policies. Through a literature review, we identified 32 studies which investigated key metrics, such as cannabis consumption, healthcare-related service use, crime, traffic crashes/fatalities, suicidal behaviors, and other drug use. Due to our stringent methodological criteria, all included studies in the review were performed in the United States of America. Overall, the evidence illustrates a lack of effect of RML on adolescent and young adult populations, and a possible increase in service use, vehicle related crashes and fatalities, and alcohol consumption. The data has not signaled an increase in nicotine use; however, it does correlate with a decrease in opioid prescriptions. It is also important to highlight the dearth of research with controlled designs related to the impact of recreational legalization of marijuana on criminality (excluding drug possession-related crimes), as well as deaths due to opioid overdoses or suicide.

To date, the evidence suggests moderate increases in past-month cannabis use in adult populations and no increase in adolescents or young adults (11). These data illustrate two central points. First, the lack of clearly detrimental effects seen in adolescence and early adulthood years is important considering that one of main concerns that was raised prior to RML was that such policy change could contribute to the development of ancillary impairments caused by increased cannabis use during early periods of brain maturation (51, 52). Second, the observation of increased consumption in adults is based on one single study which met the aforementioned methodological criteria. There is therefore a need to replicate these results in future research. As well, the results yielded from current studies refer to past-month use, which is an outcome that cannot differentiate between adult populations that are occasionally experimenting with cannabis from populations that are transitioning from occasional use to heavy use or cannabis use disorder. Early work by Montgomery et al. (53) has discerned potential increases in newly onset cannabis use in the adult population following RML, but not the underage population, suggesting heightened experimenting among adults who may not have otherwise tried cannabis, however these findings should be replicated before deemed as conclusive (53).

The data included in this review which evaluated frequent past-month cannabis use, and past-year CUD prevalence, across the age groups, was mainly extracted from a single study, and thus caution must be exerted when interpreting the findings. Nonetheless, the preliminary evidence points to increased frequent use, and CUD prevalence in the adult population. This evidence could potentially indicate a heightened rate of transition from occasional use to problematic use; acute monitoring of the situation is warranted in future studies. Among the research articles that did not meet the inclusion criteria (i.e., not comparative and/or longitudinal studies), the collected evidence is heterogeneous however does point to a potential increase as well [for a review, see (54)]. In the young adult population, the authors found no evidence of increased frequent use or problematic use, which may suggest limited enduring effects in this age group. Interestingly, in youth, authors have failed to establish altered frequent past-month consumption, however early evidence from the comparative longitudinal study by Cerdà et al. (10) highlights a heightened prevalence of past-year CUD. As cannabis potency has been continuously increasing over the last decades (55) is posited to be associated with increased adverse health outcomes, and as heavy cannabis use is associated with more harm to psychological and physical health than occasional use (56). Comparative and longitudinal studies on this issue are required in the future to evaluate the enduring impact of recreational cannabis legalization on youth marijuana consumption and health.

With respect to post-legalization trends of motor vehicles crashes, the evidence is mixed, however indicates potential increases. Specifically, the studies encompassing early adopter regions, such as Colorado, Washington, Oregon, and Alaska have shown increases in traffic crashes/fatalities (33, 38). Other studies including a vaster range of states show more divergent effects (39, 40). It may be posited that the differing modalities of RML may be associated with differential effects, or that states which legalized recreational cannabis at a later time point learned from the experiences of states which had legalized recreational cannabis earlier on (57). Nonetheless, when drawing upon the evidence generated by non-comparative or non-longitudinal studies, patterns of increases also emerge (58–63). Careful surveillance of this key metric in future research is recommended to fully grasp the weight and extent of the impact of RML.

Service use trends more readily demonstrate increases, and were predominantly related to cannabis-related hospitalizations, however divergent trends were noted among youth, with one study demonstrating increases (34), and a second study yielding decreases in hospitalizations (35). Reasons for hospitalizations may vary substantially from one individual to another, so future studies will need to disentangle these differences. Otherwise, similar populations, locations, and timeframes were utilized to study this outcome, and it is difficult to determine at this time why opposing trends surfaced from the data.

Prior to the legalization of recreational cannabis use, certain assumptions were formulated about the anticipated impact of these types of policies on the use of other substances. On the one hand, it was hypothesized that the legalization of cannabis could lead to an increase in the co- use of other substances, presumably through a mechanism of cross-sensitization (64). Others proposed, on the contrary, that by legalizing cannabis, consumers would be less exposed to organized crime to obtain the substance, thus potentially discouraging additional access of other substances through this illicit point of contact (65). Finally, other authors, inspired by the theory of self-medication, postulate that by making cannabis more accessible, consumers could substitute their consumption of other substances by turning to cannabis (66). According to our review, we observe, in the case of tobacco, an absence of change in consumption, whereas in the case of alcohol, 3 out of 7 studies have shown an increase in the consumption of this substance. A lack of studies of non-prescription opioids does not allow for any concluding remarks to be made at this time. The reasons why we denote opposing effects of legalization on tobacco and alcohol are difficult to ascertain. In the future, research should focus on alcohol consumption, which remains, to this day, one of the substances with the highest social, economic and health impacts (67, 68).

One of the most robust associations observed in this systematic review is the correlation between RML and prescription opioids. Specifically, of the five studies which investigated the effects of RML on opioid prescription patterns, none reported a significant increase; only one reported a lack of effect; and the remaining five studies reported decreases in MMEs and number of prescriptions. These RML data parallel and align with previous medical marijuana legalization data, which report decreases in the number of opioid prescriptions provided to patients; the number of prescriptions filled by patients; the number of prescriptions discontinued early by patients; MMEs prescribed to patients, etc. (48, 69, 70). Most research articles included on this topic were evaluated as having high-quality evidence. As such, the evidence is sufficient to establish a potentially beneficial association between recreational marijuana legislation and prescription opioid patterns. Influencing prescription practices and restricting access to opioids are two public health strategies which have already been implemented by the Center for Disease Control (CDC) to contain the opioid epidemic (71); one may speculate that the reduction in prescriptions denoted in the current review may be accounted for by these strategies (72). However, the comparative nature of the articles retained in this review suggest that RML states find greater reductions in opioid prescriptions compared to non-RML locations, indicating that RML status may be contributing to a synergistic effect and amplifying these efforts. Beyond this general observation, future research should clarify the nature of this relationship. For instance, it remains to be determined if there are subgroups of healthcare practitioners or organizational services (i.e., surgeons, emergency medicine physicians, family physicians; hospital, community services, etc.) that are more strongly changing their prescription habits, and if there are subcategories of patients who are targeted by these declining practices (i.e., cancer patients, patients undergoing surgeries requiring pain management care, patients with chronic pain, etc.). Likewise, it remains to be determined if the changes in opioid prescription are directly related or not to the providing of alternatives to patients (i.e., medical cannabis prescriptions). Finally, it must be noted that a causational relationship has not yet been established between RML status and opioid prescription patterns.

While a changes in opioid prescription was observed, no effects of cannabis legalization were observed on opioid-related deaths. One can hypothesize that downstream cascading effects may require lengthier follow-up periods to capture differences. Alternatively, the lack of effect on mortality despite the decrease in opioid prescriptions could be explained by the fact that most opioid-related deaths are due to the consumption of particularly powerful opioids (i.e., fentanyl) procured outside of clinical settings. Nonetheless, it is important to note that the lack of effect on opioid-related deaths is based on a single comparative and longitudinal study. Data derived from research which was not selected as a part of this review show diverging patterns, exhibiting patterns of increases, lack of effects, and decreases (73, 74). Finally, it is worth mentioning that most studies on opioid-related outcomes failed to account for significant confounders such as policies related to the delivery of overdose healthcare services, and access to overdose treatment, including naloxone and buprenorphine, which may directly impact opioid-related outcomes (75).

It is important to know that few studies corresponding to the above-reference inclusion and exclusion criteria investigated criminal activity (outside drug-relate possession crimes); evidence from non-comparative or non-longitudinal nature are conflicting, positing increases and decreases in crimes such as violent crime, property crime and sexual assaults (65, 76, 77). Similarly, only two longitudinal and comparative studies investigated the impact of RML on suicide, and none evaluated cannabis potency. Regarding the potency of cannabis, it has been steadily increasing decades before the enactment of any cannabis regulations, transitioning from an approximate 2% of delta-9-tetrahydrocannabinol (Δ^9^-THC) in 1970, to close to 15% in 2016 (55). Stronger potency of Δ^9^-THC content in cannabis products is vital to monitor, as it is most likely the main component responsible for the psychological, cognitive and health harms of cannabis (78, 79). We found no comparative and longitudinal study that has evaluated Δ^9^-THC potency changes before, and subsequent to, RML implementation. To fully assess the consequences of recreational cannabis regulations on public health, it will be relevant to assess this outcome in the future.

Despite the narrow inclusion criteria of this review, it is relevant to compare current findings with population-level health data derived from other adult (recreational) regulatory frameworks, such as the ones in Uruguay and Canada. The Uruguayan experience of recreational legalization has yielded preliminary results which are largely in accordance with the present review, with noted potential increases in the prevalence of adult use, a lack of effect on use in youth, a lack of effect on other drug use, an increase in service use such as hospital visits for intoxication, as well as an increase in serious crimes such as homicides and traffic fatality rates (80–83). Despite the recent recreational legalization in Canada, several publications have yielded crucial insights to the impacts of RML on population health. Echoing most findings from the present publication, the primary evidence suggests adult consumption is on the rise, however CUD prevalence remains stable (12, 84); RML is associated with mixed, yet potentially minimal impacts on consumption in youth (85), and may be linked with possible increases in service use, such as emergency department visits or unintentional cannabis intoxications (86–88). Vast efforts are still ongoing across both nations to better grasp the implications of recreational legalization on public health outcomes.

## 5. Limitations of the current systematic review

The strengths of the studies collected in this systematic review include their longitudinal study design, which captures important temporal variations of outcomes; and that all studies included one or more comparator locations, which controls for diverging trends occurring outside of cannabis legislation policies. Despite these strengths, a few limitations should also be noted. First, there is a lack of longitudinal comparative studies to investigate key populational health outcomes, and stronger efforts in elucidating these outcomes are required to allow for informed policy decisions. For instance, no controlled study specifically examined the effects of RML on cannabis-related mental health outcomes. Second, the implementation of the comparator criteria entailed the exclusion of all studies derived from Canadian settings. In Canada, cannabis has been legalized across all provinces, thus makes it impossible to carry out studies with comparators locations. It is possible that the trends observed in the United States may not be representative of the Canadian experience of legalization, as there are notable differences in legalization modalities between countries. For example, the Canadian experience of recreational legalization is more standardized across regions than the US experience, is overall more restrictive in terms of licensing, home growing and possession, but more liberal in terms of age of consumption, location of consumption, and limits for driving under the influence (89). Nonetheless, the data extracted from Canadian settings seems to largely parallel findings from the United States, except for consumption data, demonstrating a mixed effect RML on daily cannabis use, whereas US data suggests a likelier increase in daily use (10, 84), and service use, with once again mixed effects in Canada, and more suggestive increasing patterns in the US (90, 91).

There are several confounding factors which are infrequently controlled for, though should be accounted for when analyzing the implications of RML on public health outcomes. For example, the proliferation of marketing strategies of edibles (92) mounted alongside several reports of non-compliant tactics, especially regarding youth (93, 94) may be contributing to an uptick in adverse public health outcomes, such as pediatric exposures to cannabis or emergency department visits (95, 96). Another notable confounder in the landscape of recreational legalization is its potential “spillover effect” to neighboring non-legalized states (19, 97). This could potentially give rise to the under-estimation of effects between legalization states, especially if these neighboring states are included as comparator locations in the analyses. In addition, several studies included in the current review did not differentiate between the legalization and the delayed enactment of recreation cannabis policies; this is a crucial variable to consider in future research, as prior data has already shown a correlation between the number of outlets opened and the prevalence of consumption (98, 99). Several studies analyzed data timepoints less than one-year post-enactment–thus limiting the ability to identify patterns which either require a lengthier time to be detected or identifying patterns which do not endure in time. Finally, the legalization of medical marijuana has rendered it more difficult to scrutinize the consequences of recreational legalization, as the evidence has shown medical legalization influences public attitudes, opinions, and behaviors (100, 101).

## 6. Conclusion

Considering the entirety of the collected evidence, RML is preliminarily associated with increases in adult consumption of cannabis–but not youth consumption; however, little data from controlled studies is available on frequent/problematic cannabis use. RML is also linked to potential increases in service use, as well as traffic crashes and fatalities. Due to the lack of evidence, we could not determine any patterns associated to crimes and suicide. A potential increase in alcohol use has been observed, while no differences were observed in the case of nicotine. Interestingly, the data demonstrated a reduction of opioid prescriptions in RML states compared to non-RML states. We cannot determine if this effect yields an overall benefit or risk to mortality or morbidity of at-risk populations and thus should be a key focus for future research. Another gap in the field is the lack of controlled studies on the potential impact of RML on mental health outcomes. Finally, further research is clearly needed on the differences in RML policies.

## Data availability statement

The original contributions presented in the study are included in the article/supplementary material, further inquiries can be directed to the corresponding author.

## Author contributions

SP and MA conceptualized the systematic review and wrote the manuscript. MA and IZ performed the search and extracted the information. AD and IZ provided critical comments. All authors approved the final version of the manuscript.
